# Low-Temperature Self-Healing Cement Mortar Enabled by Novel Composite Microcapsules: Performance, Mechanism, and Optimization

**DOI:** 10.3390/ma19050933

**Published:** 2026-02-28

**Authors:** Yao Li, Yonggang Deng

**Affiliations:** School of Architecture and Civil Engineering, Liuzhou Institute of Technology, Liuzhou 545616, China; 13998802511@163.com

**Keywords:** low-temperature, microcapsule, self-healing, strength repair ratio, concrete

## Abstract

While self-healing concrete shows promise for infrastructure repair, its effectiveness is significantly compromised in low-temperature environments because of slowed reaction kinetics and the embrittlement of capsule shells. To address this limitation, novel composite microcapsules featuring an ethyl cellulose shell and a dual-core comprising expansive cement and epoxy resin were developed. These microcapsules were fabricated using a physical spheronization-coating method and subsequently incorporated into cement mortar. Response surface methodology was employed to identify the optimal system, which balances self-healing performance with the retention of mechanical properties: a microcapsule content of 3% (by mass of cement) and a particle size range of 1.4 to 1.7 mm. Under conditions of −20 °C, the optimal formulation achieved a crack surface healing ratio of up to 44.1% and a compressive strength recovery of up to 6.0%. Microstructural and spectroscopic analyses (SEM-EDS, XRD) revealed a synergistic healing mechanism. This mechanism involves the formation of calcium carbonate, C–S–H gel, and anorthite, all cohesively bonded within a polymerized epoxy network. This work establishes a functional material strategy for enabling autonomous crack repair in concrete structures subjected to cold climates. In such environments, even marginal strength recovery, when coupled with effective crack sealing, can significantly enhance structural durability.

## 1. Introduction

Concrete, the most widely used construction material globally, is inherently quasi-brittle and prone to cracking. In cold regions, freeze–thaw cycles are a potent accelerator of concrete deterioration. The cyclic freezing and thawing of pore water generates internal tensile stresses that exceed the concrete’s tensile strength, leading to progressive microcracking. These microcracks degrade mechanical properties and provide ingress pathways for aggressive agents (e.g., chlorides and sulfates), which can trigger reinforcement corrosion and substantially shorten the service life of structures [1,2,3]. Traditional repair methods, such as epoxy injection, are often ineffective, costly, and impractical in low-temperature environments where material properties and interfacial bonding are severely compromised [4,5,6]. Recent studies on the bioprotection of cementitious materials in soil environments have demonstrated that indigenous microbial activity can promote mineral precipitation and crack sealing [7]. This finding underscores the critical role of environmental factors—such as temperature, moisture, and microbial presence—in autonomous healing processes.

In response, the concept of self-healing concrete, inspired by biological systems, has emerged as a promising strategy for autonomous crack remediation [8,9,10]. Among various approaches, microencapsulation technology—pioneered by White et al. for polymers—has been extensively adapted for cementitious systems [11]. When a crack propagates, embedded microcapsules rupture and release healing agents (e.g., polymers, minerals, or alkalis) into the crack plane, thereby enabling autonomous repair [12,13,14]. Previous studies have demonstrated impressive healing efficiencies under standard curing conditions (approximately 20 °C). For instance, sodium silicate-based microcapsules have achieved complete closure of cracks up to 0.3 mm wide [15,16], while epoxy-containing capsules have restored significant fracture toughness [17,18].

However, a critical and often overlooked challenge is the drastic performance decline of these self-healing systems under low-temperature service conditions. Two fundamental limitations arise: (1) Kinetic retardation: The gelation, polymerization, and hydration reactions of most healing agents are thermally activated. At temperatures near or below 0 °C, reaction rates plummet, severely delaying or inhibiting the healing process. For example, the gelation rate of sodium silicate decreases by 73% at 5 °C compared to standard conditions [19,20,21]. (2) Capsule integrity compromise: Common polymeric shell materials (e.g., urea-formaldehyde) undergo significant embrittlement and an increased elastic modulus at low temperatures [22,23,24]. This reduces their probability of fracturing upon crack intersection and impedes the timely release of healing agents [25,26]. Consequently, the promising healing efficiencies reported under standard laboratory conditions seldom translate to reliable performance in real-world cold-climate applications. This discrepancy creates a significant gap between material design and practical utility. This situation highlights a fundamental design mismatch: most self-healing systems are optimized for standard curing conditions (approximately 20 °C) but fail to account for the coupled material-science challenges posed by sustained cold—namely, shell embrittlement and core reaction arrest. To bridge this gap, a holistic microcapsule design strategy must concurrently address both issues at sub-zero temperatures. This necessity constitutes the core motivation for the present study.

To bridge this gap, a holistic microcapsule design must concurrently address shell embrittlement and core inactivation at sub-zero temperatures. Guided by this principle, a novel composite microcapsule featuring three integrated design elements was designed: (1) An ethyl cellulose (EC) shell, selected for its retained fracture susceptibility at low temperatures, in contrast to the pronounced embrittlement of common formaldehyde-based resins [22,23,24]. (2) A dual-component core comprising epoxy resin and expansive Portland cement, designed to initiate a sequential and synergistic healing process: rapid polymer adhesion followed by sustained mineral precipitation. (3) A suite of optimized auxiliary agents to ensure manufacturability, storage stability, and functional reliability of the capsules.

Therefore, the objectives of this study are threefold: (1) to fabricate and characterize the proposed ethyl cellulose (EC)-shell/composite-core microcapsules; (2) to systematically evaluate their effects on the mechanical properties and, more critically, on the low-temperature (−20 °C) self-healing performance of cement mortar, thereby identifying the optimal microcapsule content and particle size; and (3) to elucidate the microstructural evolution and chemical mechanisms governing the healing process through integrated SEM-EDS and XRD analyses. This work aims to establish a new material paradigm for enhancing the durability and extending the service life of concrete infrastructure in cold regions.

## 2. Materials and Methods

### 2.1. Raw Materials

#### 2.1.1. Cementitious Materials and Aggregates

Portland cement (P.O 42.5, Shenyang, China) was used as the primary binder. Its chemical composition and physical properties are summarized in Table 1 and Table 2, respectively. Natural river sand (Shenyang, China), with a fineness modulus of 2.44, served as the fine aggregate. The sand was washed and dried prior to use.

#### 2.1.2. Chemicals for Microcapsule Synthesis

The composite microcapsules comprised an ethyl cellulose shell and a dual-component core consisting of epoxy resin (E51) and expansive Portland cement. Microcrystalline cellulose and hydroxypropyl methylcellulose were employed as excipients, while Tween 80 served as a surfactant. The shell-coating solvents were a mixture of toluene and anhydrous ethanol in a 4:1 volume ratio. An epoxy curing agent (T31) was incorporated into the mortar mixtures. The specifications and proportions of the core and shell materials are detailed in Table 3 and Table 4.

### 2.2. Preparation of Composite Microcapsules

#### 2.2.1. Formulation Design and Optimization

The composition of the composite microcapsules was meticulously designed to meet the dual requirements of shell integrity and core reactivity at low temperatures. The dual-core system employs an approximate 1:1 mass ratio, specifically comprising 34 wt% epoxy resin and 32 wt% expansive Portland cement. This ratio was determined through preliminary screening tests to optimize the synergy between the rapid adhesive bonding provided by the curing epoxy network and the sustained mineral precipitation resulting from cement hydration and carbonation. A higher epoxy proportion was found to potentially inhibit cement activation within confined cracks, whereas an insufficient epoxy proportion compromised the critical early-stage crack bridging at low temperatures.

The auxiliary agents were selected based on their specific and complementary functional roles in both microcapsule fabrication and performance. Microcrystalline cellulose (MCC, 30 wt%) serves as a porous, absorbent matrix that hosts the core particles and provides essential structural stability during the extrusion-spheronization process. Hydroxypropyl methylcellulose (HPMC, 2 wt%) was incorporated as a binder and plasticizer to enhance the cohesiveness and spheronization efficiency of the wet powder mixture. Furthermore, Tween 80 (2 wt%), a non-ionic surfactant, was added. It proved essential for achieving a homogeneous dispersion of the hydrophobic epoxy resin within the predominantly hydrophilic cement/MCC matrix, thereby ensuring a uniform distribution of the healing agents.

Ethyl cellulose (EC) was selected for the capsule shell in preference to more common but brittle polymers, such as urea-formaldehyde, due to its documented retention of toughness and its lower glass transition temperature (T_g_ below 0 °C. A 10 wt% EC solution was prepared in a toluene/anhydrous ethanol mixture (4:1, *v*/*v*). Toluene, as a good solvent for EC, enables the formation of a continuous film, whereas the more volatile co-solvent, ethanol, promotes rapid drying. This rapid drying prevents solvent penetration that could prematurely activate the water-sensitive cement core.

#### 2.2.2. Preparation Procedure of Composite Microcapsules

**Figure 1:**
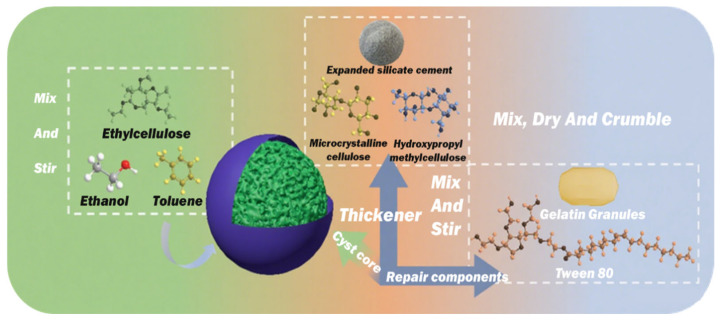
Flowchart illustrating the fabrication process of the composite microcapsules.

The composite microcapsules were fabricated via a five-step physical method that combines extrusion–spheronization with solvent coating, as illustrated in Figure 1. First, epoxy resin particles were prepared by emulsifying epoxy resin E51 in an aqueous gelatin solution. The emulsion was then solidified by cooling and subsequently ground into particles. These particles were then dry-mixed with expansive Portland cement, microcrystalline cellulose, and hydroxypropyl methylcellulose in the proportions detailed in Table 3. A surfactant solution (Tween 80 in deionized water) was then incorporated under continuous mixing to form a homogeneous wet mass. The wet mass was then processed through an extrusion-spheronization system. In this system, the mass was first extruded into cylindrical strands, which were subsequently rounded into spherical pellets by centrifugal force on a rotating plate. The resulting pellets were then coated by spraying them with an ethyl cellulose solution (dissolved in a toluene/ethanol mixture, 4:1 *v*/*v*) within a rotating drum to form a continuous polymeric shell. Finally, the coated microcapsules were sieved to isolate three specific particle size fractions (0.85–1.18 mm, 1.18–1.4 mm, and 1.4–1.7 mm) for subsequent study. This sieving step also removed defective or irregular particles.

### 2.3. Preparation of Cementitious Composites

Mortar specimens with a constant water-to-cement-to-sand mass ratio of 1:2:6 were prepared. The key variable was the microcapsule content, which was incorporated at 0%, 3%, 6%, and 9% by mass of cement. The content of the T31 curing agent was fixed at 50% of the microcapsule mass. The detailed mix proportions are summarized in Table 5. Specimens for compressive and flexural strength tests were cast in prismatic molds measuring 40 mm × 40 mm × 160 mm. For self-healing evaluation, pre-cracked specimens were prepared and cured under specified environmental conditions. The use of cement mortar (without coarse aggregate) conforms to standard laboratory practice for fundamental studies. This approach simplifies the mixture, ensures better control over microcapsule distribution, and facilitates clearer interpretation of the self-healing mechanism.

### 2.4. Test Method

#### 2.4.1. Mechanical and Self-Healing Performance Tests

Mechanical Strength. The compressive and flexural strengths of the cement mortar were tested at curing ages of 3, 7, and 28 days in accordance with Chinese Standard GB/T 17671-2021.

Self-Healing Evaluation: Specimens were preloaded to 70% of their ultimate compressive strength (σ_max_) to induce controlled microcracking. Because ultimate strength varies with microcapsule content, this protocol does not yield identical internal damage states across different mixtures. This preloading level was instead selected for consistently generating surface cracks with an average width of 150–250 μm, a range that provides comparable initial conditions for evaluating self-healing efficiency. Initial crack width was measured at four premarked points along the primary crack path using a digital microscope. The initial crack width was measured using a digital microscope at four pre-marked points along the primary crack path. The pre-cracked specimens were then placed in controlled environments at either a low temperature (−20 °C) or room temperature (20 °C) for healing periods of 3, 7, 14, and 28 days. The crack width was re-measured at the same marked points at each interval to monitor the healing progression. It should be noted that this method quantifies healing primarily at the specimen surface. While it serves as a widely used indicator of repair activity, it does not directly assess the extent of healing through the full crack depth or the restoration of internal mechanical continuity.

After the designated healing period, the compressive strength of the healed specimens was tested. The strength recovery ratio (η) was then calculated using Equation (1):

(1) $$\eta \;=\;\frac{F_{\mathrm{healed}}-F_{\mathrm{damaged}}}{F_{\mathrm{damaged}}}$$

where *F*_damaged_ and *F*_healed_ are the compressive strengths (MPa) of the specimen after pre-damage and after the healing period, respectively. The strength recovery ratio (η) quantifies the net recovery of compressive strength attributable specifically to the autonomous crack-healing process. By using the pre-damaged strength (F_damaged_) as the baseline, this metric isolates the contribution of the released healing agents. It thereby excludes any concurrent strength gain from the continued hydration of the intact cement matrix. The crack-width surface healing ratio (ω) was calculated using Equation (2):

(2) $$w_{\mathrm{recover}}\;=\;\frac{A_{0}-A_{n}}{A_{0}}\times 100\%$$

where *A*_0_ is the initial average crack width (mm), and *A*_n_ is the average crack width after *n* days of healing. The crack-width surface healing ratio (*w*) is defined as the percentage reduction in the visible crack width at the specimen surface, reflecting the physical filling and sealing of the crack. This metric directly quantifies the crack-closing efficiency of the healing products. It is a surface-specific indicator of repair activity and does not necessarily represent the extent of healing through the full crack depth or the recovery of internal mechanical continuity.

A four-factor, three-level Central Composite Design (CCD) within the framework of Response Surface Methodology (RSM) was employed to systematically investigate the effects of curing temperature (A), curing duration (B), microcapsule content (C), and particle size (D) on the crack healing ratio. The experimental design, run order generation, and subsequent statistical analysis were performed using Design-Expert^®^ software (version: 13.0.5). The CCD is suitable for fitting a second-order polynomial model to capture potential linear, interaction, and quadratic effects of the factors.

#### 2.4.2. XRD Analysis

X-ray diffraction (XRD) was employed to characterize the phase composition of the specimens and the healing products. The measurements were performed using Cu Kα radiation with a scanning speed of 10°/min over a 2θ range of 5° to 90°.

#### 2.4.3. SEM Analysis

To investigate the healing products and underlying mechanisms, scanning electron microscopy coupled with energy-dispersive X-ray spectroscopy (SEM-EDS) was performed on the fracture surfaces of healed specimens. Samples were extracted from the crack zones of specimens healed for different periods at −20 °C.

## 3. Results and Discussion

### 3.1. Characterization of the Microcapsules

#### 3.1.1. Morphological Analysis

**Figure 2:**
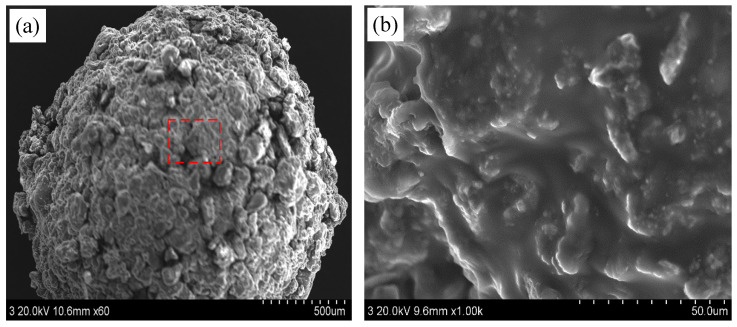
SEM images of the microcapsule morphology: (**a**) overall distribution and (**b**) a magnified view of the boxed region in (**a**), revealing the surface texture.

The surface morphology of the prepared microcapsules was characterized using scanning electron microscopy (SEM), as shown in Figure 2. The microcapsules exhibited a generally spherical shape with a slightly rough surface texture (Figure 2a). Higher-magnification imaging (Figure 2b) revealed that the surface was uniformly coated with a dense layer of cellulose-based material. This dense, coherent coating is anticipated to preserve the microcapsule integrity during mixing and incorporation into the cementitious matrix [27,28].

#### 3.1.2. Chemical Structure Analysis

**Figure 3:**
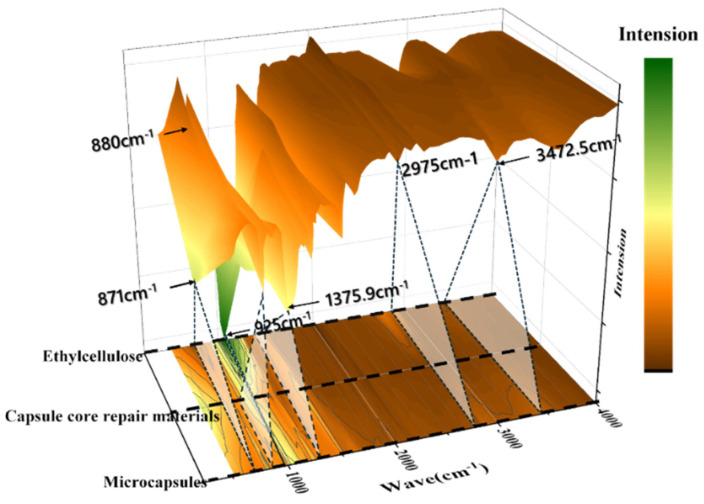
Fourier-transform infrared (FTIR) spectra of the shell material, core mixture, and final composite microcapsules.

Fourier-transform infrared (FTIR) spectroscopy was employed to verify the successful encapsulation of the core materials within the ethyl cellulose (EC) shell. The FTIR spectra of the individual shell material (EC), the core material mixture, and the final composite microcapsules are presented in Figure 3.

The FTIR spectrum of the composite microcapsules exhibits characteristic peaks of both the shell and core materials, confirming successful encapsulation. Characteristic peaks of ethyl cellulose are observed at 3472 cm^−1^ (O–H stretching), 2975 cm^−1^ (C–H stretching of methyl and methylene groups), and 1376 cm^−1^ (symmetric CH_3_ bending). In addition, weak bands at 925 cm^−1^ and 880 cm^−1^ are attributed to vibrations of the ethyl groups. The presence of the core materials is indicated by a distinct peak at 871 cm^−1^, which corresponds to the Si–O stretching vibration of the expansive cement component. The coexistence of these characteristic peaks in the microcapsule spectrum confirms the structural integrity of the core–shell composite [29,30].

### 3.2. Mechanical Properties

**Figure 4:**
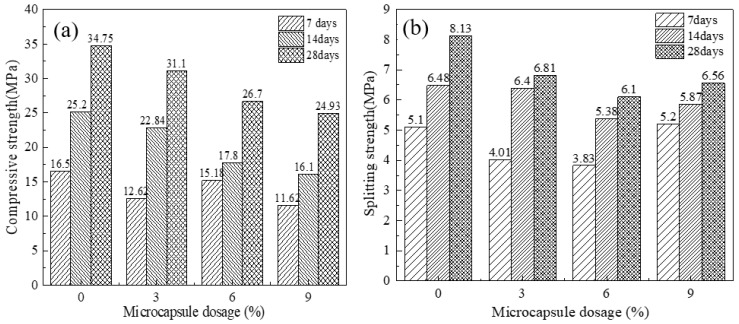
Mechanical properties of microcapsule-modified cement mortar (**a**) Compressive strength and (**b**) Flexural strength.

The compressive and flexural strengths of all mixtures were measured at curing ages of 3, 7, and 28 days, with the mixture containing 0% microcapsules serving as the control. The results are presented in Figure 4. As shown in Figure 4a, compressive strength increased with curing age for all mixtures but decreased with increasing microcapsule content, reaching a minimum at a content of 9%. This reduction is attributed to the microcapsules displacing strength-contributing cementitious material and introducing weak interfaces within the matrix.

As shown in Figure 4b, flexural strength exhibited a similar general decreasing trend as microcapsule content increased from 0% to 6%. Interestingly, the mixture with 9% microcapsules exhibited higher early-age (3-day) strength. This can be attributed to a filler effect that enhanced the compactness of the fresh mixture prior to substantial matrix hydration. However, at 28 days, the flexural strengths of the mixtures containing 3%, 6%, and 9% microcapsules were 16.2%, 24.9%, and 19.3% lower than that of the control, respectively. This result confirms the overall strength-reducing influence of the incorporated microcapsules.

Notably, the strength reduction for the 9% mixture (19.3%) is less severe than that for the 6% mixture (24.9%). This non-monotonic trend suggests that at higher dosages, additional mechanisms may partially counteract the strength loss. Two contributing factors are hypothesized. First, the increased quantity of ductile ethyl cellulose shell fragments within the matrix could act as discrete, crack-bridging elements that deflect and blunt microcracks during flexural loading. Second, a limited autogenous healing effect might occur. Even in uncracked specimens, a small fraction of microcapsules may rupture due to mixing stresses or early-age shrinkage. The released epoxy and cementitious agents could then seal inherent micropores and microcracks during the 28-day curing period. This self-sealing effect would be more pronounced in the 9% mixture owing to the higher microcapsule concentration. This could potentially result in a denser and less flawed matrix at 28 days compared to the 6% mixture. Although the net effect of microcapsule incorporation remains a reduction in absolute strength, the balance between these potentially positive mechanisms and the dominant negative effect of interface weakening appears to shift at the 9% dosage.

### 3.3. Evaluation of Repair Effectiveness

#### 3.3.1. Effect of Microcapsule Particle Size on the Strength Recovery Ratio

**Figure 5:**
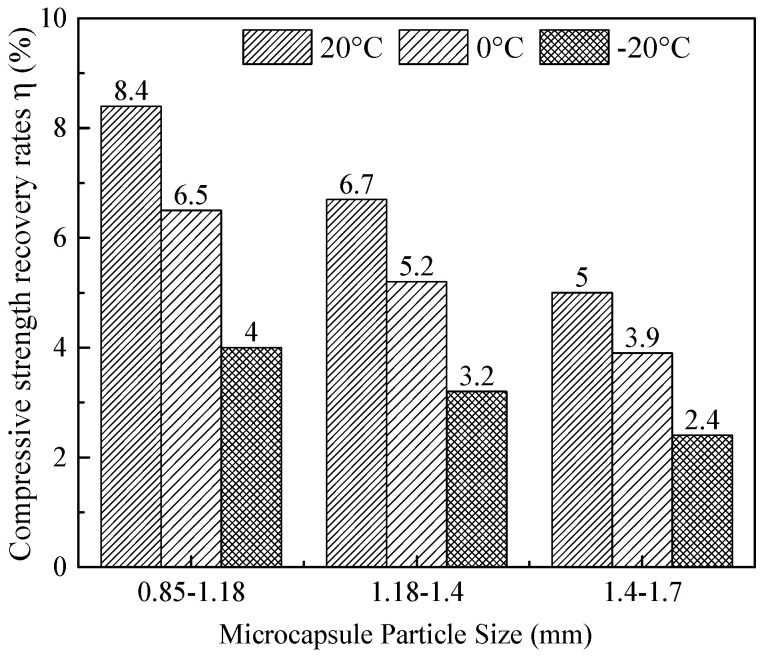
The strength recovery ratio of mortar specimens incorporating 3% microcapsules, as influenced by microcapsule particle size and curing temperature.

Figure 5 illustrates the strength recovery ratio of cement mortar specimens incorporating 3% microcapsules, with three distinct particle size ranges (0.85–1.18 mm, 1.18–1.4 mm, and 1.4–1.7 mm), after 7 days of self-healing at 20 °C, 0 °C, and −20 °C. The specimens were preloaded to 70% of their ultimate compressive strength (σ_max_) prior to healing.

At all three temperatures, the strength recovery ratio increases monotonically with decreasing microcapsule particle size. For instance, at 20 °C, the ratio rises from 5.0% for the largest size range (1.4–1.7 mm) to 8.4% for the smallest (0.85–1.18 mm). At −20 °C, it increases from 2.4% to 4.0% over the same particle size ranges. This trend is attributed to two interrelated factors. For a given mass content, smaller capsules offer a higher number density and greater total reactive surface area [30]. Consequently, they provide a higher probability of crack–capsule intersection and more efficient release of healing agents upon cracking [31,32,33].

#### 3.3.2. Effect of Microcapsule Content on the Strength Recovery Ratio

**Figure 6:**
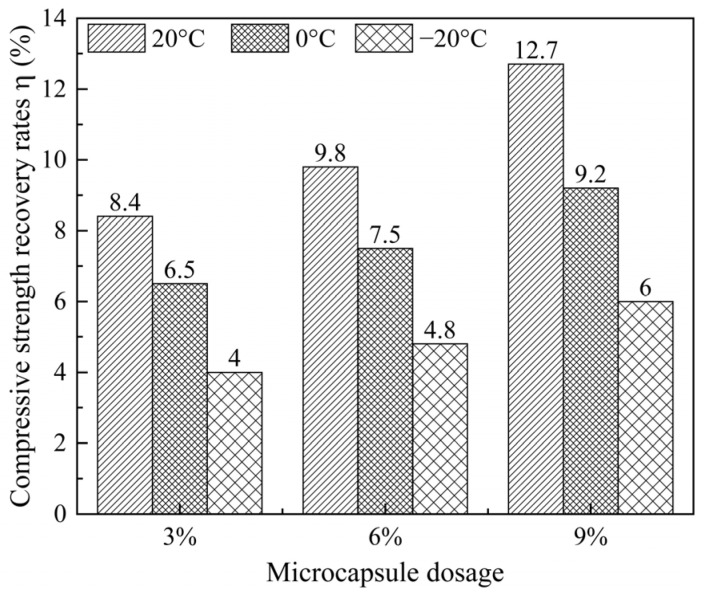
Influence of microcapsule content and curing temperature on the strength recovery ratio.

Figure 6 shows the strength recovery ratio of cement mortar specimens as a function of microcapsule content (3%, 6%, and 9%) and curing temperature (20 °C, 0 °C, and −20 °C). For a given microcapsule content, the strength recovery ratio decreases substantially with decreasing temperature. At 20 °C, the ratios are 8.4%, 9.8%, and 12.7% for 3%, 6%, and 9% content, respectively; at 0 °C, they fall to 6.5%, 7.5%, and 9.2%; and at −20 °C, they decline to 4.0%, 4.8%, and 6.0%. This temperature dependence reflects the thermally activated nature of epoxy polymerization and cement hydration, the two key healing reactions [19,20,21].

Moreover, at each curing temperature, the strength recovery ratio increases monotonically with microcapsule content. The 9% mixture consistently yields the highest recovery of the three, even at −20 °C (6.0%). This positive correlation arises from the larger reservoir of healing agents supplied by higher capsule dosages, which enables more extensive crack filling and interfacial bonding upon release [34,35]. Despite the overall reduction in healing efficiency at sub-zero temperatures, the system retains a measurable self-healing capability, demonstrating the effectiveness of the proposed microcapsule design.

#### 3.3.3. Effect of Microcapsules on Crack-Width Healing

The experimental data from the orthogonal array (Table 6) were fitted to a second-order polynomial model using coded factors (Equation (3)). The corresponding analysis of variance (ANOVA) is presented in Table 7. The model is statistically highly significant (*p* < 0.0001) and demonstrates excellent predictive capability. This is evidenced by a high coefficient of determination (R^2^ = 0.9912) and the close agreement between the adjusted R^2^ (0.9758) and predicted R^2^ (0.9741). This model provides a quantitative framework for elucidating the complex, non-linear interactions that govern the low-temperature healing process [36,37].

w = 60.18 + 4.31A + 22.79B + 26.68C + 6.70D + 0.36AB + 1.45AC + 5.18AD + 2.80BC − 1.97BD − 3.72CD − 6.50A^2^ − 4.48B^2^ − 9.60C^2^ − 54.79D^2^ (3)

where A, B, C, and D are the independent factors in coded units (−1, 0, +1), corresponding to the factor levels detailed in Table 8.

A critical insight from the ANOVA is the dominant, statistically significant effect of the quadratic term for microcapsule content (C^2^), which has the largest F-value (215.08). This identifies microcapsule content as the most influential factor, acting in a non-linear manner. This non-linearity fundamentally captures the intrinsic trade-off in capsule-based self-healing systems. While increasing the microcapsule content supplies a greater reservoir of healing agents—thereby enhancing the crack-filling potential—it simultaneously introduces more weak interfacial zones and dilutes the cementitious matrix [34]. The optimal microcapsule content was determined to be approximately 3%. This value represents the point that maximizes the net healing benefit, achieving the best compromise between enhancing autonomous crack repair and preserving the intrinsic mechanical properties of the cementitious matrix. Therefore, while higher microcapsule contents (e.g., 6% or 9%) can provide greater crack-sealing capacity, this comes at the expense of a more significant reduction in mechanical strength.

**Figure 7:**
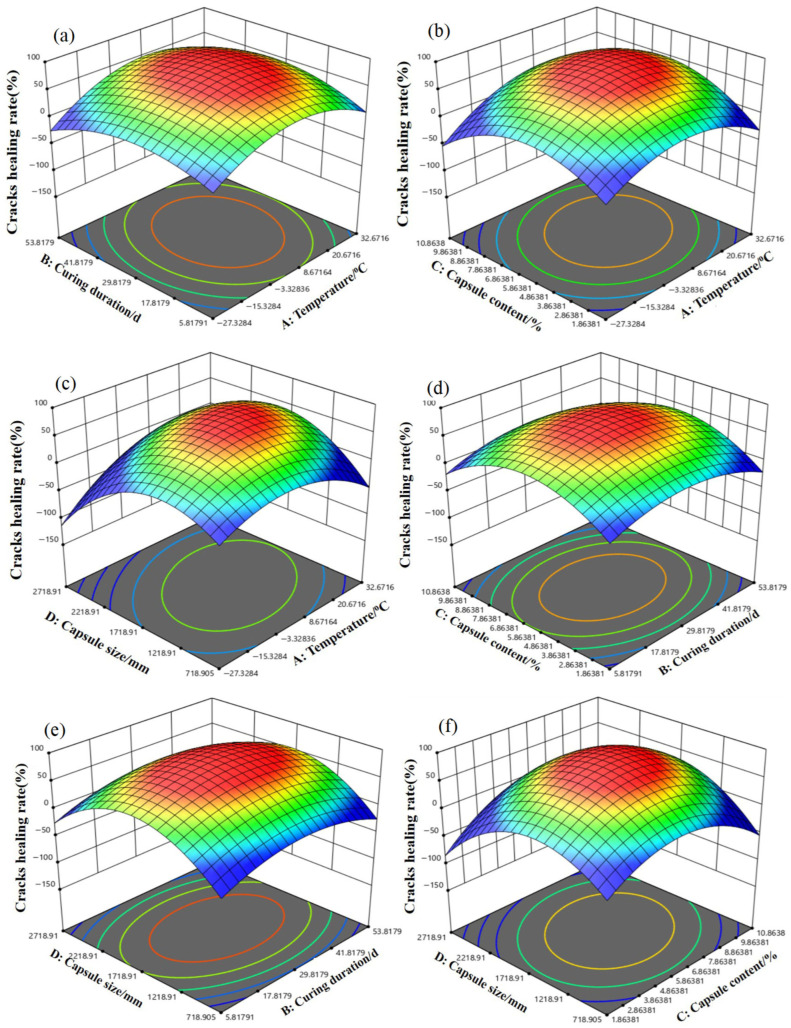
Three-dimensional diagram of response surfaces of multi-factor interactions. (**a**) Effect of curing duration and temperature on crack healing rate (**b**) Effect of capsulecontent and temperature on crack healing rate (**c**) Effect of capsule size and temperatureon crack healing rate (**d**) Effect of capsule content and curing duration on crack healingrate (**e**) Effect of capsule size and curing duration on crack healing rate (**f**) Effect of capsulesize and capsule content on crack healing rate.

Furthermore, the analysis reveals a statistically significant interaction between curing temperature and duration (AB interaction term, *p* = 0.0462). The nature of this interaction, visualized in the corresponding 3D response surface plot (Figure 7), has important practical implications. This interaction indicates that the effect of curing duration on the healing ratio is strongly temperature-dependent. Specifically, at sub-zero temperatures (e.g., −20 °C), achieving a substantial healing ratio requires a considerably longer curing period compared to ambient conditions. This finding directly corroborates the kinetic limitations that low temperature imposes on key healing reactions, such as epoxy polymerization and cementitious hydration [38]. Consequently, for field applications in cold climates, designs must account for an extended “healing time window.” This requirement challenges the performance expectations derived from standard laboratory curing protocols.

In contrast to the strong non-linear and interactive effects described above, most two-factor interaction terms (e.g., AC, AD, BC) were not statistically significant. This suggests that, within the studied parameter ranges, factors such as microcapsule content and particle size can be optimized with relative independence, provided that the critical temperature–duration relationship is first addressed. The linear term for particle size (D) was statistically significant. This aligns with the monotonic improvement in healing performance observed for smaller particles (Figure 5), which is attributable to their higher number density and greater reactive surface area.

Several interaction and quadratic terms (AC, AD, BC, BD, CD, D^2^) were not statistically significant (*p* > 0.05; see Table 8). However, in accordance with the hierarchical modeling principle, they were retained in the final model (Equation (3)) to preserve model hierarchy, maintain interpretability, and ensure the model adequately represents the full experimental design domain. Consequently, the following discussion focuses on the significant and dominant effects, specifically the C^2^ and AB terms.

The response surface model successfully identifies the optimal parameter combination, which centers on a microcapsule content of ~3%, a smaller particle size, and extended curing at low temperature. Furthermore, it elucidates the fundamental principles governing the system. Specifically, the model quantifies the compromise between healing agent volume and matrix integrity, underscores the kinetic necessity for prolonged healing periods in cold environments, and provides a validated tool for tailoring the self-healing system to specific low-temperature service conditions.

Using the validated regression model (Equation (3)), numerical optimization was performed to identify the factor combination that maximizes the crack healing ratio (*w*). The desirability function approach was employed, with the goal set to maximize ω while constraining all factors within their experimental ranges. For standard conditions (20 °C), the model predicts a theoretical maximum healing ratio of approximately 75.5% for a curing duration of 28 days, a microcapsule content of about 5.4%, and a minimal particle size (~1.0 mm). To address the core objective of low-temperature self-healing, a constrained optimization was conducted by fixing the curing temperature (A) at −20 °C. Under this constraint, the model identifies the optimal combination as follows: a curing duration (B) of 28 days, a microcapsule content (C) of approximately 3.3%, and a particle size (D) at the range 1.4–1.7 mm. This model-driven result robustly supports the empirical finding that a microcapsule content of approximately 3% is optimal for low-temperature performance. Furthermore, it specifies the concomitant optimal curing duration and particle size.

### 3.4. Microstructures Analysis

**Figure 8:**
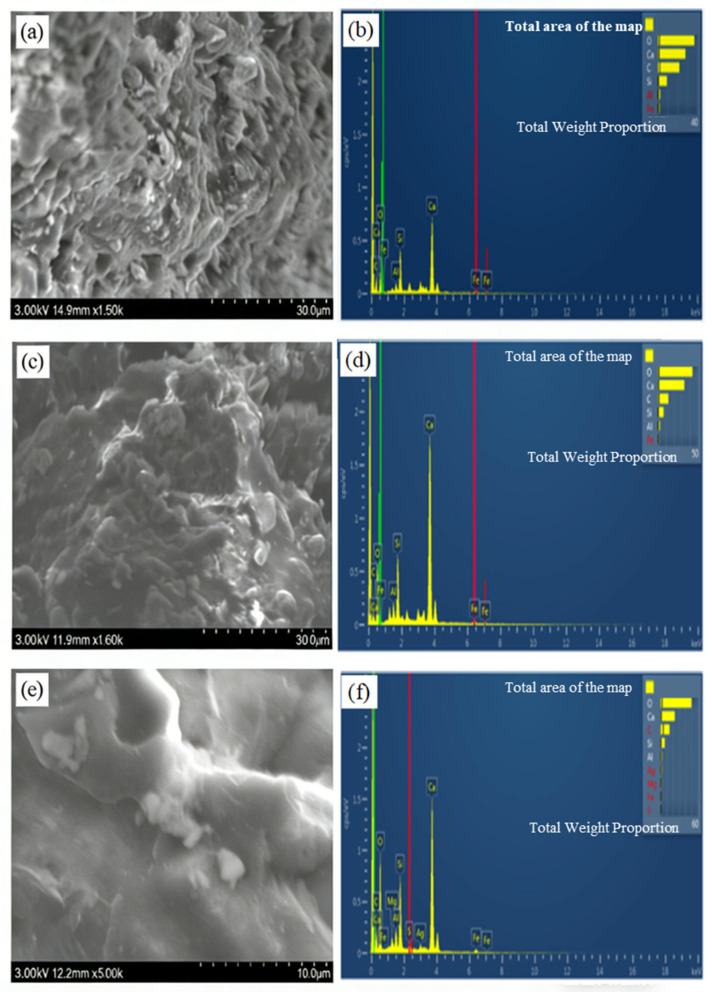
SEM-EDS Analysis of Repair Products in Microcapsule-Modified Cement Mortar after 7 d Healing at −20 °C (**a**,**b**) 3%, (**c**,**d**) 6% and (**e**,**f**) 9%.

After 7 days of healing at −20 °C, the microstructural evolution and chemical composition of repair products in cement mortar specimens with 3%, 6%, and 9% microcapsules were examined using SEM-EDS (Figure 8).

At 3% microcapsule content (Figure 8a), the crack-filling material appears as discrete white particulates with a rough, discontinuous morphology. This observation indicates limited deposition of healing agents and corresponds to the modest strength recovery ratio measured for this mixture. The corresponding EDS spectrum (Figure 8b) is dominated by O, Ca, C, and Si, indicating that the primary repair phases are calcium carbonate (CaCO_3_) and calcium silicate compounds, which likely originate from carbonation of portlandite and pozzolanic reactions.

At 6% microcapsule content (Figure 8c), the repair layer becomes more coherent and compact, featuring blocky crystalline deposits and a smoother surface with numerous protrusions. This morphology reflects enhanced crack-filling efficiency. The EDS profile (Figure 8d) reveals a minor Al peak alongside O, Ca, C, and Si, indicating the formation of a chemically more diverse assemblage, including CaCO_3_, calcium silicate hydrate (C–S–H), and traces of an aluminum-bearing phase, likely anorthite (CaAl_2_Si_2_O_8_).

At the highest microcapsule content of 9% (Figure 8e), the crack is completely bridged by a dense, adhesive-like white matrix with a uniform and smooth surface. This morphology indicates effective sealing and strong interfacial bonding. The EDS spectrum (Figure 8f) remains consistent with those of lower-content specimens, showing dominant O, Ca, C, and Si signals. However, the marked improvement in microstructural continuity suggests that increasing capsule content does not introduce new chemical phases but substantially enhances the volume and cohesion of the key repair products (CaCO_3_, C–S–H gel, and anorthite).

Collectively, these observations demonstrate a content-dependent transition from sparse, particulate deposits to a continuous, dense repair matrix. This microstructural evolution directly explains the macroscopic healing efficiency: the sparse CaCO_3_ particles observed at 3% content correspond to the lower strength recovery ratio, whereas the cohesive, C–S–H and epoxy-rich matrix at 9% content underlies the superior crack sealing and strength recovery. The consistent detection of carbonate and silicate-based phases across all microcapsule contents confirms that the healing mechanism is governed by the synergistic hydration of expansive cement and curing of epoxy resin.

### 3.5. X-Ray Diffraction (XRD) Analysis

**Figure 9:**
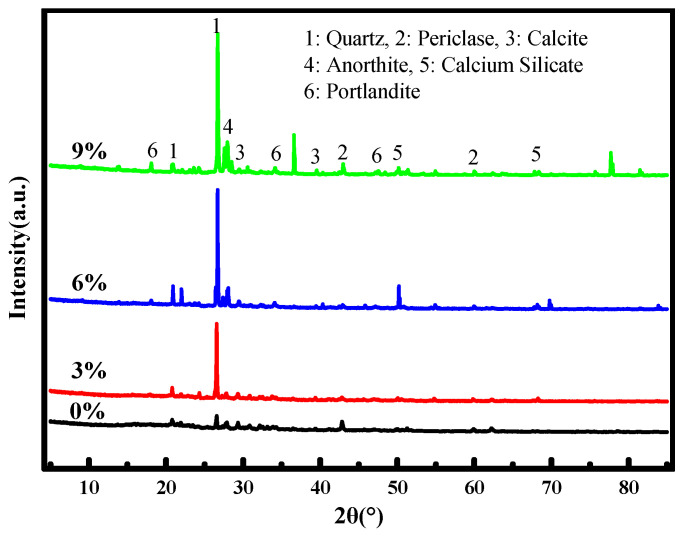
X-ray diffraction (XRD) patterns of the repair products extracted from crack surfaces in cement mortar specimens containing 0%, 3%, 6%, and 9% composite microcapsules after 7 days of self-healing at −20 °C.

X-ray diffraction analysis was performed to identify the crystalline phases present in the repair products extracted from the crack zones of cement mortar specimens after 7 days of healing at −20 °C (Figure 9).

In all diffractograms, the most intense peaks correspond to quartz (SiO_2_), which originates from the fine aggregate (river sand) unavoidably included during sampling. Beyond this background signal, several distinct crystalline phases related to the healing process are identified. A pronounced peak near 17° 2θ is assigned to portlandite (Ca(OH)_2_), a hydration product of Portland cement that serves as a reactant reservoir for subsequent carbonation. The characteristic peak of calcite (CaCO_3_) appears at approximately 29° 2θ, indicating carbonation of portlandite by atmospheric CO_2_—a key crack-sealing mechanism. A well-defined peak around 28° 2θ is attributed to anorthite (CaAl_2_Si_2_O_8_), suggesting the reaction of cement constituents with water, CO_2_, and available calcium hydroxide. Diffraction signals near 52° and 68° 2θ are associated with various calcium silicate phases, reflecting the presence of incompletely hydrated cement grains within the crack zone. Although amorphous calcium silicate hydrate (C–S–H) gel does not produce sharp XRD peaks, its formation is inferred from the overall reaction context and is consistent with the SEM-EDS observations.

**Figure 10:**
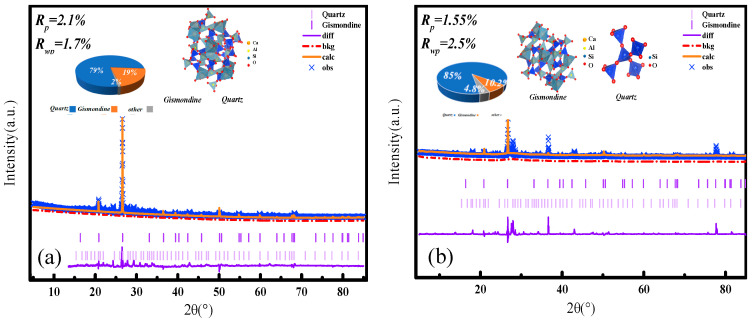
Rietveld refinement of XRD patterns for the cement mortar specimen containing 9% microcapsules after healing at −20 °C for (**a**) 28 days and (**b**) 7 days. The refinements highlight the evolution of the quartz peak position and the formation of secondary crystalline phases.

To better understand the crystalline phase evolution during low-temperature self-healing, Rietveld refinement was performed on XRD patterns of crack-surface material. The specimens contained 9% microcapsules and were healed for 7 and 28 days at −20 °C (Figure 10). Quartz served as an internal standard to evaluate peak shifts and secondary phase formation. The goodness-of-fit indices, specifically the profile R-factor (*R_p_*) and the weighted profile R-factor (*R_wp_*), confirmed the reliability of the refinement models (Equations (4) and (5)).

(4) $$R_{p}=\frac{\;\sum_{i=1}^{n}|Y_{\mathrm{obs},i}-Y_{\mathrm{calc},i}|}{\sum_{i=1}^{n}Y_{\mathrm{obs},i}}$$

(5) $$R_{\mathrm{wp}}=\frac{\sqrt{\sum_{i=1}^{n}w_{i}\cdot (Y_{\mathrm{obs},i}-Y_{\mathrm{calc},i}{)}^{2}}}{\sqrt{\sum_{i=1}^{n}w_{i}\cdot Y_{\mathrm{obs},i}^{2}}}$$

After 28 days of healing (Figure 10a), a distinct positive shift in the quartz peak positions relative to the reference is evident. This indicates either lattice strain or overlapping diffraction from newly formed crystalline phases. Well-defined peaks corresponding to portlandite (Ca(OH)_2_) appear at 18.1°, 34.0°, and 47.1° 2θ, assigned to the (001), (101), and (110) planes, respectively. This demonstrates that cement hydration progressed substantially despite prolonged low-temperature curing. Notably, characteristic peaks of ettringite emerge at 15.8° and 22.9° 2θ, corresponding to the (202) and (220) planes, confirming the release of sulfate and aluminate ions from the expansive cement core. The crystallization of ettringite contributes to crack filling and may induce beneficial expansion that enhances sealing.

After only 7 days of healing (Figure 10b), the quartz peak shift is substantially smaller, indicating reduced lattice distortion or interference from secondary phases at this early stage. The portlandite peaks at 18.1°, 34.0°, and 47.1° 2θ are notably more intense and symmetric than those at 28 days, reflecting a higher concentration of crystalline Ca(OH)_2_ before its gradual consumption via carbonation and pozzolanic reactions. Ettringite peaks are barely discernible at 15.8° and 22.9° 2θ, confirming that ettringite formation is kinetically limited during initial healing at −20 °C.

The comparative refinement results demonstrate a time-dependent phase evolution within the repair zone. At 7 days, the system is dominated by portlandite from ongoing hydration, whereas the formation of ettringite—a key expansive repair product—remains incipient. After 28 days, consumption of portlandite and progressive crystallization of ettringite become evident. This trend aligns with the enhanced crack bridging and strength recovery observed in the mechanical tests, underscoring a dual-stage healing mechanism. This mechanism involves initial pore filling by carbonates from Ca(OH)_2_, followed by longer-term, expansive sealing via ettringite formation [39,40]. The refinement data provide crystallographic validation that the composite microcapsules successfully drive a sequential repair chemistry even under sustained low-temperature conditions, leading to microcrack closure [41].

## 4. Repair Mechanism Analysis

Commonly used shell materials—such as urea-formaldehyde (UF) and melamine-formaldehyde (MF) resins—undergo significant embrittlement and increased elastic modulus below 0 °C [22,23,24]. Such embrittlement impairs capsule rupture and delays the release of healing agents. This property can severely hinder capsule rupture and the timely release of healing agents. The ethyl cellulose (EC) shell employed in this work was deliberately selected to mitigate this issue. Regarding the healing chemistry, single-component systems (e.g., alkali silicates or pure epoxy) often face limitations such as insufficient long-term durability, incomplete volumetric crack filling, or severe kinetic retardation in cold environments. The dual-core strategy leverages the complementary strengths of organic and inorganic repair mechanisms. The epoxy resin provides immediate adhesion-based crack bridging, a process less thermodynamically hindered at low temperatures. Simultaneously, expansive cement enables long-term volumetric filling via hydration products and carbonates. Although kinetically slowed at low temperatures, this mineralization process proceeds effectively within the crack space, which is pre-stabilized by the polymerized epoxy network. This synergistic, multi-stage mechanism offers a more robust and reliable healing pathway for concrete infrastructure in cold climates than the single-mechanism systems reported previously [15,16,17,18,38].

**Figure 11:**
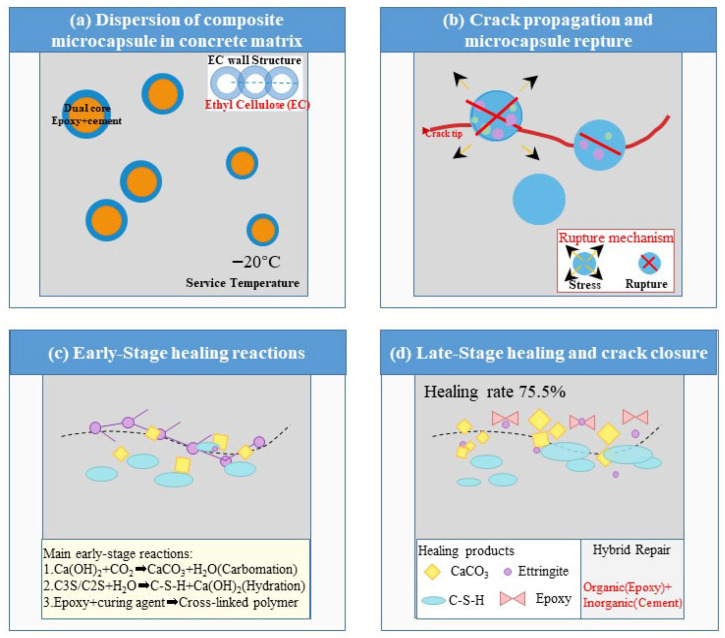
Proposed sequential self-healing mechanism of the cementitious system incorporating composite microcapsules under low-temperature conditions.

Based on the integrated experimental results, a sequential low-temperature self-healing mechanism for the composite microcapsule-modified cementitious system is proposed, as illustrated in Figure 11. The process initiates with the homogeneous dispersion of microcapsules—featuring an ethyl cellulose (EC) shell and a dual-component core of epoxy resin and expansive Portland cement—within the cementitious matrix. The EC shell retains its fracture susceptibility at −20 °C, ensuring its ability to rupture upon cracking (Figure 11a). Upon crack propagation, the intersecting microcapsules rupture due to stress concentration, thereby releasing the encapsulated healing agents into the crack plane (Figure 11b). Subsequently, during the early healing stage (approximately 7 days), the released epoxy resin polymerizes to form an adhesive network. Concurrently, cement hydration and carbonation reactions precipitate calcium carbonate (CaCO_3_) crystals and calcium silicate hydrate (C–S–H) gel, which collectively initiate crack filling (Figure 11c). This early stage is corroborated by the measurable strength recovery observed at 7 days (e.g., 6.5% at 0 °C for a 3% content; see Figure 6) and by the predominant portlandite (Ca(OH)_2_) and initial calcite (CaCO_3_) phases identified via XRD at this age (Figure 10b). Ultimately, after prolonged curing (approximately 28 days), a dense and cohesive hybrid repair matrix matures. This matrix comprises CaCO_3_, C–S–H gel, and expansive ettringite crystals, all cohesively bonded by the continuous, polymerized epoxy network (Figure 11d). The maturation of this matrix corresponds to the peak crack-surface healing ratio (up to 75.5%) and the maximum strength recovery (up to 8.4% at 20 °C), as reported in Section 3.3. It is also consistent with the enhanced crystalline phase evolution—notably ettringite formation—detected by XRD after 28 days (Figure 10a). This synergistic healing mechanism enables up to 75.5% surface crack closure at a sustained 20 °C. This visible crack filling, together with the concurrent strength recovery, confirms the continued activity of the released healing agents within the crack plane.

Leveraging this mechanistic advantage, a comparison of key performance metrics further underscores the advancement achieved in this work. Direct quantitative comparisons across studies are challenging due to variations in testing methods, crack dimensions, and healing conditions. Nevertheless, a qualitative assessment of low-temperature efficacy remains instructive. Many prominent self-healing systems reporting high healing efficiencies are evaluated at or above 0 °C [15,17,30]. For instance, studies on epoxy-based or bacterial healing often report a dramatic decline in healing activity as temperatures approach 0 °C, with some systems showing negligible activity below 5 °C [19,25]. In this context, the present system achieves a strength recovery of 6.0% and a crack-surface healing ratio of 44.1% under sustained −20 °C conditions (Table 7, Figure 6). These values represent a measurable functional capability under severe cold, whereas many alternative systems exhibit markedly reduced or negligible performance at such temperatures. While few studies report quantitative healing data at similarly low temperatures, the results presented here establish a performance benchmark for a material system that is both specifically engineered for and rigorously validated under severe cold-climate conditions. This performance stems from the integrated design of a low-temperature-ductile shell and a synergistic dual-core healing system. It directly addresses the critical need for reliable autonomous repair in cold-climate concrete infrastructure.

## 5. Conclusions

To address the challenges of low efficiency and limited crack-size adaptability exhibited by existing self-healing systems in cold climates, this study developed a novel composite microcapsule. This microcapsule features an ethyl cellulose shell and a dual-core system comprising expansive cement and epoxy resin. The main findings are as follows:

(1) An optimal microcapsule content of 3% (by mass of cement) was identified. This content balances effective self-healing performance with minimal compromise to the mechanical strength of the mortar. Higher microcapsule contents (6% and 9%) further enhance crack-sealing capacity but lead to a more significant reduction in compressive strength. Under sustained −20 °C conditions, the system achieved a maximum compressive strength recovery of 6.0% (at 9% content) and a surface crack-healing ratio of 44.1%. At 20 °C, the mixture with a 3% microcapsule content yielded a strength recovery of 8.4%. The response surface model predicts that with optimized curing parameters, crack-healing ratios of up to 75.5% are attainable.
(2) Microstructural and spectroscopic analyses confirmed a synergistic healing mechanism. This mechanism involves the precipitation of calcium carbonate (CaCO_3_) and calcium silicate hydrate (C–S–H) gel, alongside the formation of anorthite, all cohesively bonded within a continuous polymerized epoxy network. An optimal microcapsule particle size range of 1.4–1.7 mm was identified. This range ensures a high probability of rupture upon crack intersection while minimizing the detrimental internal stress concentrations induced by larger particles within the cementitious matrix.
(3) A sequential low-temperature healing mechanism was elucidated. This mechanism integrates three key stages: capsule rupture upon cracking, early adhesive bonding provided by the polymerized epoxy, and long-term mineral precipitation from cement hydration and carbonation. Collectively, this work provides a practical and efficient material strategy. This strategy is designed to enhance the durability and extend the service life of concrete infrastructure in cold regions by enabling autonomous crack repair under sub-zero temperatures.

## Figures and Tables

**Table 1:** Chemical composition of cement (%).

Component	CaO	SiO_2_	Al_2_O_3_	Fe_2_O_3_	MgO	R_2_O	SO_3_	Loss
Content	62.97	21.18	5.61	4.07	1.74	0.51	2.45	1.47

**Table 2:** Cement physical performance index.

Specific Surface Area m^2^/Kg	Density g/cm^3^	Initial Setting Time/Min		Compressive Strength/MPa		Flexural Strength/MPa	
		Initial	Final	3d	28d	3d	28d
350	3.1	198	264	23.6	52.3	5.7	9.6

**Table 3:** Composition of the microcapsule core material.

Substance	Content (wt.%)	Specification	Materials Sources
Epoxy Resin Particles	34	Chemically Pure	Nantong Xingchen Synthetic Materials Co., Ltd. (Nantong, China)
Expanded Portland Cement	32	Chemically Pure	Guangxi Yunyan Special Cement Building Materials Co., Ltd. (Hengzhou, China)
Microcrystalline Cellulose	30	Column Chromatography Grade	Shanghai Macklin Biochemical Technology Co., Ltd. (Shanghai, China)
Hydroxypropyl Methylcellulose	2	Chemically Pure	Shenyang Cellulose Factory (Shenyang, China)
Tween 80	2	Chemically Pure	Tianjin Damoo Chemical Reagent Co., Ltd. (Tianjin, China)

**Table 4:** Composition of the microcapsule wall material and solvent.

Component	Substance	Content (%)	Specification	Materials Sources
Wall Material	Ethyl Cellulose	10	Chemically Pure	Shanghai Macklin Biochemical Technology Co., Ltd. (Shanghai, China)
Solvent	Toluene/Ethanol Mixture	90	-	-
	Toluene	80	Analytical Pure	Suzhou Jiren Advanced Materials Co., Ltd. (Suzhou, China)
	Anhydrous Ethanol	20	Analytical Pure	Tianjin Damoo Chemical Reagent Factory (Tianjin, China)

**Table 5:** Mix proportions of cement mortar (kg/m^3^).

No.	Component	0%	3%	6%	9%
1	Cement	450	450	450	450
2	Fine Aggregate	1350	1350	1350	1350
3	Water	225	225	225	225
4	Microcapsules	0	13.5	27	40.5
5	Curing agent	0	6.75	13.5	20.25

**Table 6:** Analysis of Orthogonal Experiment Results.

Number	Temperature/°C	Curing Duration/d	Microcapsule Content/%	Particle Size/mm	Cracks Healing Ratio/%
1	−20	3	6	1.18–1.4	8.2
2	20	3	6	1.18–1.4	32.4
3	−20	28	6	1.18–1.4	44.1
4	20	28	6	1.18–1.4	71.8
5	0	14	3	0.85~1.18	15.4
6	0	14	9	0.85~1.18	12.3
7	0	14	3	1.4–1.7	39.4
8	0	14	9	1.4–1.7	31.3
9	−20	14	6	0.85~1.18	15.4
10	20	14	6	0.85~1.18	46.2
11	−20	14	6	1.4–1.7	11.6
12	20	14	6	1.4–1.7	56.3
13	0	3	3	1.18–1.4	9.7
14	0	28	3	1.18–1.4	47.5
15	0	3	9	1.18–1.4	10.4
16	0	28	9	1.18–1.4	54.1
17	−20	14	3	1.18–1.4	23.4
18	20	14	3	1.18–1.4	51.2
19	20	14	9	1.18–1.4	41.9
20	20	14	9	1.18–1.4	42.4
21	0	3	6	0.85~1.18	10.6
22	0	28	6	0.85~1.18	70.1
23	0	3	6	1.4–1.7	14.3
24	0	28	6	1.4–1.7	71.2
25	0	14	6	1.18–1.4	66
26	0	14	6	1.18–1.4	55.5
27	0	14	6	1.18–1.4	54.3
28	0	14	6	1.18–1.4	55.6
29	0	14	6	1.18–1.4	64.1
30	0	14	6	1.18–1.4	54.8
31	0	14	6	1.18–1.4	63.9
32	0	14	6	1.18–1.4	65.1
33	0	14	6	1.18–1.4	65.4
34	0	14	6	1.18–1.4	65.7
35	0	14	6	1.18–1.4	54.2
36	0	14	6	1.18–1.4	49.3

**Table 7:** Analysis of Variance (ANOVA).

Source	Sum of Squares	Freedom	Root-Mean-Square	F Value	*p* Value
model	18,752.3	14	1339.45	28.34	<0.0001
A	189.4	1	189.4	4.01	0.0482
B	338.6	1	338.6	7.16	0.0091
C	4582.1	1	4582.1	96.94	<0.0001
D	215.8	1	215.8	4.56	0.0354
AB	164.2	1	164.2	3.47	0.0462
AC	19.1	1	19.1	0.4	0.5286
AD	8.6	1	8.6	0.18	0.6723
BC	28.1	1	28.1	0.59	0.4437
BD	52.3	1	52.3	1.11	0.2958
CD	22.8	1	22.8	0.48	0.4899
A^2^	3124.5	1	3124.5	66.09	<0.0001
B^2^	538.8	1	538.8	11.34	0.0012
C^2^	10,162.4	1	10,162.4	215.08	<0.0001
D^2^	28.4	1	28.4	0.6	0.4412
Residual	992.7	21	47.27		
Misfit term	874.3	10	87.43	1.85	0.1521
Net error	118.4	11	10.76		
Total	19,745.0	35			
R^2^ = 0.9912, R_Adj_^2^ = 0.9758, R_Pred_^2^ = 0.9741					

**Table 8:** Response surface experimental factors and horizontal encoding.

Factor	Horizontal Coding		
	−1	0	1
curing temperature/°C	−20	0	20
curing duration/d	3	14	28
microcapsule content/%	3	6	9
particle size/mm	0.85~1.18	1.18–1.4	1.4–1.7

## Data Availability

The original contributions presented in this study are included in the article. Further inquiries can be directed to the corresponding author.
